# The context matters - not all prolonged sitting bouts are equally related to momentary affective states: an ambulatory assessment with sedentary-triggered E-diaries

**DOI:** 10.1186/s12966-021-01170-3

**Published:** 2021-08-14

**Authors:** Martina Kanning, Christina Niermann, Ulrich Ebner-Primer, Marco Giurgiu

**Affiliations:** 1 University of Konstanz, Department of Sports Science, Social and Health Sciences, Universitätsstraße 10, 78464 Konstanz, Germany; 2 Department of Sports and Sports Science, mental mHealth lab, Hertzstraße, Karlsruhe Institute of Technology (KIT), 16, 76131 Karlsruhe, Germany; 3 Central Institute of Mental Health, Department of Psychiatry and Psychotherapy, Medical Faculty Mannheim, Heidelberg University, J5, 68159 Mannheim, Germany

## Abstract

**Background:**

Sedentary behaviors (SB) and especially prolonged sitting bouts are highly prevalent in daily life and studies indicated an association with an increased risk for several non-communicable diseases. Consequently, guidelines to reduce SB were developed. At the same time, an in-depth knowledge regarding SB such as where, what and with whom people spend time sedentary as well as correlates such as affective states of prolonged sitting bouts, is still lacking. A more differentiated view on SB is necessary to identify detrimental and modifiable sedentary bouts. We addressed this gap by conducting an ambulatory assessment study including accelerometer and sedentary-triggered e-diaries that captures data during prolonged sitting bouts (> 20 min). We investigated how contextual factors of prolonged sitting bouts are associated with momentary affective states.

**Method:**

Four studies were combined with a final sample of 308 participants (50.3% female, M_age_, 27.4, range, 17–66). SB was assessed objectively with thigh-worn accelerometers for four to five days. Whenever a participant was sitting for 20 or 30 min the accelerometer triggered questions assessing social (not alone vs. alone) and environmental (leisure vs. working) factors as well as momentary affective states (valence, energetic arousal and calmness). Multilevel analyses were used to examine within-person associations between different contexts and mood during prolonged sitting.

**Results:**

Momentary affective states varied significantly due to different social and environmental contexts (*p*_s_ < 0.001): Sitting together with others was associated with higher levels of valence and energetic arousal. Furthermore, sitting during leisure time was associated with higher levels of valence and calmness and lower levels of energetic arousal. Significant interaction analyses revealed that participants had the highest ratings while sitting during leisure episodes together with others.

**Conclusion:**

Findings showed that prolonged sitting bouts differ regarding their association with affect. Sitting with others, sitting during leisure time and especially sitting during leisure time and with others, was associated with higher levels of momentary affective states, respectively. Thus, SB guidelines should focus on reducing those SB episodes that are associated with lower levels of affect, for example during working episodes.

**Supplementary Information:**

The online version contains supplementary material available at 10.1186/s12966-021-01170-3.

## Background

Sedentary behaviors (SB) are highly prevalent in daily life. They are defined as any waking behavior characterized by an energy expenditure < 1.5METs while in a sitting or reclining posture [1]. Based on findings of studies using device-based measurements of SB, adults spend most of their waking hours (55%) being sedentary [2–4]. Furthermore, there is strong evidence from prospective observational studies, systematic reviews and meta-analysis, that SB is associated with all-caused mortality, fatal and non-fatal cardiovascular diseases, as well as with type 2 diabetes [5–8]. Especially prolonged sitting bouts (> 20 or 30 min) seem to be associated with health risks [9, 10] and such findings led to a consensus statement to increase sitting breaks [11]. All in all, sedentary behavior is receiving increasing recognition as a health related behavior and the WHO recently published new guidelines not only for physical activity but also for sedentary behavior [12] (https://www.who.int/activities/developing-new-guidelines-on-physical-activity-and-sedentary-behaviour-for-youth-adults-and-sub-populations).

However, the evidence on SB is preliminary and incomplete. Firstly, even though evidence about associations between SB and physical health outcomes is strong, more studies addressing the associations between sedentary behavior and mental health (e.g. emotional, psychological, and social well-being) are needed [13]. Existing evidence focused mainly on depression. A meta-analysis reported positive associations between an increased risk of depression and SB in cross-sectional, and longitudinal studies [14]. A further meta-anlyses confirmed these findings but indicate a significant associations only in femals but not in males [15]. Secondly, as Emmanuel Stamatakis and colleagues [16] pointed out, findings on health reducing effects of SB are in many cases based on correlational studies that used self-report measures of sedentary behavior (e.g. time spend sedentary or time watching TV) or waist-worn accelerometers, operationalizing sedentary behavior as time without ambulatory movement, that are incapable to differentiating between sitting and standing posture [17] Furthermore, studies using device-based measures mostly use total sedentary time and focus on the between-subject perspective of the sedentary behavior - mental health link. They do not examine fluctuations of the behavior itself and do not assess time-varying covariates, such as different settings in which sedentary behavior occurs. Thus, there is a lack of evidence how settings (e.g. leisure vs. work), days (e.g. weekday vs. weekend day), or social context factors (e.g. being with others vs. being alone) of SB are associated with (mental) health. A meta-analysis including twelve prospective cohort studies showed positive associations between the risk of depression and SB [18]. Interestingly, subgroup analysis showed that mentally passive SB such as watching TV was significantly associated with risk of depression, whereas mentally active sedentary behavior such as using a computer was not. To advance guidelines and inform the development of effective interventions, we need to know more about context and type specific associations between SB and indicators of mental health such as subjective well-being and affective states aiming to identify those sedentary episodes that are negatively associated with mental health. As device-based measures of SB do not capture situational contexts or types of sedentary behavior, studies combining device-based measures with self-report are warranted [13].

This approach, namely using smart connected e-diaries with wearables, is most often labeled by the umbrella term Ambulatory Assessment (AA). Although other terms have been used for this kind of methodology too, like ecological momentary assessment [19] (EMA) or experience sampling [20], they have in common the use of computer-assisted methodology to assess self-reported symptoms, behaviors, or physiological processes, while the participant undergoes normal daily activities [21]. Importantly, AA allows researchers to gather data near real-time that minimizes recall bias and to assess data in real-life which enhances ecological validity [22, 23]. The combination of objectively measured behavior and real-time assessment of self-reported concomitant factors enables researchers to gain in-depth knowledge regarding individual and environmental correlates of different SB episodes [23].

This study applied an AA including a device-based measure (thigh-worn) of SB and a sedentary-triggered e-diary of contextual factors and momentary affect to investigate the link between prolonged sitting bouts and momentary affective states in different social and environmental contexts.

Previous studies using different types of AA showed that objectively assessed SB is associated with lower positive affect in daily life: In a 15-day diary study [24] 121 women were prompted 4 times a day. SB was associated with less positive affect at the same time and more sedentary minutes at one occasion were associated with less positive affect at the next occasion. There was no significant association between SB and negative affect. Giurgiu and colleagues found negative associations between SB and the two affect dimensions valence and energetic arousal in a five day AA-study with 10 e-diary occasions per day [25]. Another study assessing working adults six times a day for three consecutive days analyzed lagged associations in both directions (reciprocal relations) [26]. Neither affective valence predicted subsequent SB, nor SB predicted subsequent affective valence. However, higher than usual affective arousal was associated with less subsequent SB. In sum, these AA-studies indicated that SB is negatively associated with positive affective states and concluded that SB should be reduced to strengthen positive affect or - the other way around - recommended to strengthen positive affect to help (working) adults to avoid sedentary behavior in everyday life.

Affective states refer to core affect, which is defined as “the most elementary consciously accessible affective feelings” [27]. Feelings and emotional well-being refer to mental health and could be assessed in more discrete (e.g. emotions, depression) or dimensional forms (e.g. core affect) [28]. Furthermore, they could be measured as a general construct (on a trait level) or as a momentary construct (on a state level). With respect to emotional well-being, the frequency of positive affective states is more important than their intensity [29] This study used a dimensional approach to capture affective states and differentiate between valence (i.e. pleasant vs. unpleasant), energetic arousal (high vs. low), and calmness (relaxed vs. agitated). The feelings on these three dimensions are simple, therewith not reflective or attributed to any cause [30]. They represent assessments of one’s current condition and allow us to capture and compare feelings of different sedentary situations in its natural metric.

Due to the above-mentioned findings, could we conclude that every sedentary episode is negatively associated with affective states? Isn’t it more likely that different sedentary episodes are differently associated with affectives states? Firstly, SB is not a homogenous behavior, it is multifaceted: it occurs during work, leisure-time, household work, or transport. Secondly, Gardner and colleagues questioned that SB itself is a meaningful action and showed that it is merely a procedural subcomponent of actions performed while seated such as working, talking, driving, or reading [31]. Romanzini and colleagues [32] described SB in different contextual situations in 124 young adults. The authors conducted an E-diary-study with eight to nine random alarms per day during seven consecutive days. The results showed that SB occurred mainly at home (46.3%), followed by work (32.7%). When young adults were at home, they were mainly watching TV and during working situations, they were mainly using a tablet or computer, followed by reading. Concerning social context, being alone was related to SB in nearly half of the situations. However, this study and to our knowledge no other study analyzed intraindividual variations between different contexts of sedentary and furthermore, how these context situations are associated with affective states.

We performed the present study to examine the interplay of environmental and social context factors, prolonged sitting bouts and affective states. We applied an objective state-of-the-art measure of sedentariness, namely accerelative devices fixed to the thigh, b) an EMA method (E-diary) to gather information on momentary affective states as well as contextual factors without any retrospective distortions, and c) combined both via a sedentary trigger to capture affective experiences exactly during those behavioral episodes of interest - long sitting bouts, d) a within-subject analysis approach to investigate situations and not persons, combining four studies with more than 300 participants and 6000 sedentary bouts. The purposes of the analyses are to investigate how different social contexts (not-alone vs. alone) as well as different environmental contexts (leisure vs. work) are associated with three different affect dimensions (valence, energetic arousal, calmness) during prolonged sitting bouts. We controlled for several moderating factors such as length of sitting bouts, time of day, day of week as well as for gender and age.

## Methods

### Participants

Participants from four independent studies were selected. All participants were recruited within the university setting (i.e., employees and students). Study 1 (*N* = 57; May to August 2017) and 2 (*N* = 106; September 2019 to March 2020) occurred at the Karlsruhe Institute of Technology (KIT), Germany. Study 3 (*N* = 76; May to July 2019) and 4 (*N* = 69; January to March 2019) occurred at the University of Konstanz, Germany. We selected university employees and students because they are at an increased risk of engaging in high levels of SB [33, 34]. Only participants without restrictions in performing their daily activities (i.e., those without injury or disease) were included in the study. The final sample consisted of 308 participants (50.3% female), with a mean age of 27.4 years (range: 17–66) and a mean body-mass index (BMI) of 22.8 kg/m^2^ (range: 16.3–32.4; for details see Table 1). In all studies, participants received oral and written information regarding the study procedures before written informed consent was obtained. Moreover, participants were instructed to use the study smartphone and were fitted with accelerometers.

**Table 1:** Participants characteristics

	Study 1 (n = 57; 5 d^**1**^)	Study 2 (n=106; 5 d^**1**^)	Study 3 (n =76; 4 d^**1**^)	Study 4 (n =69; 4 d^**1**^)
**Variable**	**Mean ± SD (Min-Max)**	**Mean ± SD (Min-Max)**	**Mean ± SD (Min-Max)**	**Mean ± SD (Min-Max)**
**Age [yrs.]**	34.6 ± 9.9 (25-62)	23.4 ± 5.9 (17-57)	28.6 ± 11.6 (19-66)	26.3 ± 8.5 (21-60)
**Sex [female %]**	56.1%	55.7%	52.9%	47.4%
**BMI [kg/m**^**2**^**]**	22.9 ± 3.1 (17.7-32.1)	22.3 ± 2.1 (16.3-27.1)	23.5 ± 3.0 (17.1-32.4)	not assessed
**Total sedentary bouts**^**a**^	5.4 ± 2.7 (1.8-15.6)^b^	2 ± 0.5 (1-3.4)^b^	8.7 ± 4.6 (1.5-20.5)^c^	6.4 ± 3.78 (1-17)^c^
**Valence [1-6]**^**a**^	4.6 ± 0.6 (3.2-6)	4.6 ± 0.8 (2.5-6)	4.8 ± 0.7 (2.9-6)	4.4 ± 0.7 (2.5-6)
**Calmness [1-6]**^**a**^	4.8 ± 0.7 (2.9-6)	4.7 ± 0.8 (2.5-6)	4.6 ± 0.6 (2.3-6)	4.5 ± 0.6 (3-5.9)
**Energetic arousal [1-6]**^**a**^	4.2 ± 0.7 (2.6-5.8)	3.8 ± 0.8 (2.1-5.8)	4.1 ± 0.6 (2.4-5.4)	3.6 ± 0.7 (2.3-5.8)
**Social context [alone %]** ^**a**^	45.1 ± 23.7 (0-95.8)	46.2 ± 31.4 (0-100)	38.3 ± 27.8 (0-100)	46.4 ± 25.4 (0-100)
**Environmental context [Work %]** ^**a**^	86.3 ± 18.5 (22.2-100)	49.3 ± 29.8 (0-100)	31.2 ± 29.6 (0-100)	65.6 ± 26.5 (0-100)

^1^ number of monitoring days per study

^a^ aggregated within study day per participant

^b^ sedentary bouts of 30 minutes

^c^ sedentary bouts of 20 minutes

### Ambulatory assessment

In all four studies, we used an AA to capture within-person associations between SB and momentary affective states. Although each study had some individual differences concerning procedures and study settings (see Table 2), all studies used the same measurements and are based on the same technical equipment. In particular: a thigh-worn accelerometer (i.e., Move accelerometer; movisens.com), an electronic diary (i.e., the application movisensXS on a smartphone with Android operating system), and a technical interface between the e-diary and accelerometer (i.e., Bluetooth Low Energy (BLE)) for real-time assessment. In simple terms, the thigh-worn accelerometer analyzed data on body position (sitting/lying or standing) and transferred the momentary value of the body position in real-time to the smartphone. Each time an uninterrupted, specified amount of sitting/lying posture was recorded; an e-diary was triggered on the smartphone to assess real-time context information and momentary affective states. In Study 1 and 2 the e-diary questions were triggered when participants remained in a sedentary bout of >/= 30 min, whereas study 3 and 4 implemented a sedentary trigger time of 20 min. We introduced in an earlier study the sedentary triggered e-diary approach and identified it as an accurate method for collecting contextual information during sedentary bouts. In particular, we found that over 80% of all occurred sedentary bouts were detected by our used algorithm [35].

**Table 2:** Study characteristics and E-diary items

	Study 1	Study 2	Study 3	Study 4
**Duration [days]**	5	5	4	4
**Days**	Wednesday-Sunday	Wednesday-Sunday	Thursday-Sunday	Monday-Thursday
**Participants**	57	106	76	69
**Accelerometer [wear position]**	Thigh worn move 3	Thigh-worn move 4	Thigh-worn move 3	Thigh-worn move 3
**Smartphone**	Motorola Moto G	Nokia 6	Motorola Moto G	Motorola Moto G
**Sampling Scheme**	Mixed sampling (Random and Triggered e-diaries)	Mixed sampling (Random and Triggered e-diaries)	Triggered e-diaries	Triggered e-diaries
**Sedentary trigger**	30 min	30 min	20 min	20 min
**Time out phase**	Minimum: 40 min Maximum: 100 min	50	20 min	20 min
**Prompt frequency**	Unlimited	Maximum: 6 per day	Unlimited	Unlimited
**EMA items**				
**Mood**	Visual analog scale (0-100)	Visual analog scale (0-100)	Likert scale (1-6)	Likert scale (1-6)
**Environmental context (response options)**	*Where are you currently?* (home, work, restaurant, shopping, bus/train, leisure activities, family members, at friends/partners, doctor appointment, exercise, other)	*Where are you currently?* (At home, workplace, leisure, transport)	*Where are you currently?* (workplace, canteen, at home, restaurant, bus/train, car, other)	*To which domain would you assign your current sedentary activity?* (work, leisure, home, transport)
**Social context (response options)**	*Are you alone at the moment?* (yes, no)	*Are you alone at the moment?* (yes, no)	*With whom?* (alone, colleagues, friends, family, strangers,other)	*With whom?* (alone, colleagues, friends, family, strangers, other)

### Procedure

Participants of all four studies were invited via mail and were trained at the study labor to answer the questions of the electronic diary on a loaned study smartphone (i.e., Motorola Moto G, Motorola Mobility LLC (studies 1, 3, and 4) and Nokia 6, Nokia Corporation, Espoo, Finland, nokia.com (study 2) during their visit. Furthermore, participants of all four studies got verbal and written instructions how to place the accelerometer. Participants were asked to carry the electronic diary with them during waking hours. E-diary data were stored on the smartphone till the end of measurement time and were then wirelessly uploaded on a secure server in Germany by research staff. Participants were prompted several times during waking hours (study 1 and 2: 7:30 am to 10 pm; study 3 and 4: 6:00 am to 10 pm) after they had been sitting for 30 min (study 1 and 2) or 20 min (study 3 and 4). Frequency and time out phases differed between studies (see Table 2).

### Measurements

#### Sedentary behavior in daily life

Three studies used a thigh-worn Move 3 accelerometer and one study used a thigh-worn Move 4 accelerometer. Both devices have the same technical capabilities, i.e., a single-unit accelerometer that captures movement acceleration and body positions with a range of ±16 g at a sampling frequency of 64 Hz (movisens GmbH). Raw acceleration was stored on an internal memory card. The Move accelerometer has been shown to be a valid device for recording movement acceleration and body positions [17, 36]. Participants from study 2 wore the accelerometer during the entire measurement period, whereas participants from study 1,3, and 4 removed the sensor during sleep, swimming and taking a shower. After data collection, the recorded raw acceleration data were processed in 1-min intervals by using the manufacturers’ software DataAnalyzer (v.1.13.5; 1.13.7). During this step, a bandpass filter (0.25 to 11 Hz) automatically eliminated gravitational components or artifacts (e.g., sensor shocks or vibrations when cycling on a rough road surface). Non-wear epochs were identified via the proprietary software and set as missings for further analyses. As main parameter, we processed body postures (i.e., sitting/lying or standing) in 1-min epochs.

#### Affective states

We used a three-dimensional construct to assess affective states and used the Short Mood Scale, which is the only instrument that has been explicitly developed and evaluated for use in AA-studies [37]. The bipolar scale contains six items that assess the intensity of each affect dimension: *valence* (unwell vs. well, discontent vs. content), *calmness* (relaxed vs. tense, calm vs. agitated), and *energetic arousal* (tired vs. awake, without energy vs. full of energy). Participants responded on the prompt “At this moment, I feel …” by moving a slider from the left end (e.g., unwell) to the right end (e.g., well) of the bipolar scale in study 1 and 2. In study 3 and 4 the participants answered on a 6-point likert scale. Since study 1 and 2 used a visual analog scale (0–100), the values were transferred into a 6-point likert scale as used in study 3 and 4 by dividing the 0–100 scale into six parts (e.g., 0–16.67 resulted in value 1). Scores for each subscale (valence, calmness, and energetic arousal) were obtained by averaging the corresponding two item scores. Cronbach’s Alpha ranged between 0.71 and 0.76 in our sample.

#### Social and environment context

During each E-diary prompt, participants indicated their location (e.g. home, mensa, …), what they were currently doing (e.g. working, reading, eating, ...) and if anyone was with them (no one, friends, family, …). Because options for answering were not exactly the same in all four studies (see Table 2), for the current analysis, two dichotomous variables were created. To build an indicator for environmental context three authors (CN, MG, MK) rated independently if the situation during E-diary prompts refer to leisure (0) or to working (1) episodes. In 94.3% of 6157 situations, authors received consensus, in remaining situations agreement was received after discussion. To create an indicator for the social environment, we subsumed all situations in which the participants were together with someone else as “not alone” (1) and contrasted this with situations of being “alone” (0).

#### Covariates

Five variables were used to assess moderating effects. First, to control for different study procedures, length of sitting bouts were included as a dummy variable with 30 min as the reference group. Second, we controlled for diurnal variations in the three affect dimensions while including time of day and controlled for the linear and squared effects of time. Third, we assessed if weekday or weekend is associated with affect dimensions. In the fourth and fifth place we statistically controlled for gender and age.

### Statistical analysis

Prior to the analyses, we merged 1-min values of the accelerometers and the e-diary entries using DataMerger, version 1.8.0 (movisens.com). In our final data set, we included only sedentary triggered prompts, if the participants had worn the accelerometer within the 20 or 30 min prior to the prompt (i.e., > 86% of all prompts). Moreover, we included random prompts from study 1 and 2, if the participants remained in an uninterrupted sitting bout. To test our hypotheses, i.e., within-person effects of social and environmental context on affective states while remaining in sedentary bouts (≥ 20 min), we conducted multilevel analyses [38]. Multilevel analysis has several advantages, such as (i) the analysis of hierarchically structured data (i.e., multiple assessments nested within participants), (ii) robustness concerning missing data points, and (iii) separate within- and between-person effects. In particular, we conducted two-level-models and nested repeated measurements (level 1) within participants (level 2). First, intraclass correlation coefficients (ICCs) were estimated using unconditional models including valence, energetic arousal, and calmness as outcomes to separate the variance into within- and between subject sources. Second, we added the time-invariant and time-variant predictors age [yrs], sex [female vs. male], length of bouts [20 min. vs. 30 min.], time [hours], time-squared [hours^2^], day of week [weekday vs. weekend day], social context [not-alone vs. alone] and environmental context [leisure vs. work] to our models. For exploratory analyses, we added the level-1 interaction between social and environmental context as a further covariate into our model. Overall, we conducted a total of six models. As an example of one final model, the equation is presented below. All multi-level analyses were conducted using SPSS (version 26, IBM). We set the α level to 0.05 for all analyses (syntax is available as Additional file 1).

### Within-person analyses [hypothesis 1]

$$ Y{\left(\boldsymbol{valence}\right)}_{ij}={\upbeta}_{00}+{\upbeta}_{01}\ast {age}_j+{\upbeta}_{02}\ast {sex}_j+{\upbeta}_{03}\ast length\ {of\ bouts}_j+{\upbeta}_{10}\ast time\ of\ {day}_{ij}+{\upbeta}_{20}\ast time\ of\ {day}_{ij}^2+{\upbeta}_{30}\ast {weekday}_{ij}+{\upbeta}_{40}\ast {social\ conte xt}_{ij}+{\upbeta}_{50}\ast environmental\ conte\mathrm{x}{t}_{ij}+{u}_{0j}+{u}_{1j}\ast time\ of\ {day}_{ij}+{u}_{2j}\ast {weekday}_{ij}+{u}_{3j}\ast {social\ conte xt}_{ij}+{u}_{4j}\ast {environmental\ conte xt}_{ij}+{r}_{ij} $$

On level 1, within-person effects were estimated for participants´ (subscript _*j*_) mood ratings at any time of measurement (subscript _*i*_). *Y*_*ij*_ represents the level of valence, energetic arousal and calmness, respectively, in person *j* at time *i*. We centered valence, energetic arousal, and calmness on the participant mean. Beta coefficients represent the intercept (β_00_) and the effects of time, time-squared, day, and social and environmental context (β_1j_ − β_5j_) at level 1, and r_ij_ represents the residuals at level 1. On level 2, between-person effects were estimated. We included random effects (i.e., individual variation on the sample mean effect *γ*) for each predictor represented as μ_ij_. Random slope parameters (μ_1j_ − μ_4j_) were kept in the model only if significant (*p* < .05) variation was observed across participants. To compare the effects of each predictor, we calculated standardized beta coefficients (stand. BC) following established procedures (Hox, 2014). Moreover, to compare the model fit, we used -2ΔLL likelihood ratio test. To calculate the proportion of explained total outcome variance, we used the predicted outcome’s squared correlation (R^2^) by using the fixed effects and actual values [39].

## Results

In Table 1, the sample characteristics are detailed on a study level. Across all studies, self-reported mood via e-diary was collected during 6157 sedentary bouts (≥ 30 min in study 1 and 2 (*n* = 2349); ≥ 20 min in study 3 and 4 (*n* = 3814). Participants reported average mood scores of 3.89 (energetic arousal), 4.62 (valence), and 4.66 (calmness). The mean compliance across all studies was 58.6% (ranging from 36.5 to 86.1% on a study level). The ICCs revealed that 61% (ρI = 0.39; valence), 71% (ρI = 0.29; energetic arousal) and 63% (ρI = 0.37; calmness) of the variance in the mood ratings was due to within-person fluctuations. Participants remained in sedentary bouts on average, 5.29 ± 4.08 times per day (ranging from 1 to 20.5). In 43.7% (*n* = 2691) of all sedentary bouts, participants reported that they were alone, and 40.8% (*n* = 2514) of all bouts occurred during work time.

### Social and environmental context

#### Valence

As hypothesized, social context significantly predicted valence (*p* = < .001), i.e., sitting while being with others was associated with higher valence ratings. In particular, being alone compared to being not alone differed in ratings of valence by 0.17 units on average (scale 1–6). Furthermore, environmental context significantly predicted valence (*p* = < .001), i.e., prolonged sitting bouts in the leisure context were related to higher valence ratings. In other words, sitting while working compared to sitting while performing leisure activities differed by 0.32 units on average (scale 1–6). Furthermore, time and time-squared significantly influenced valence (all *p’s* = < .001) in both negative and positive directions. In practice, valence decreased during the day until approximately 4 pm, followed by a subsequent increase until the end of the day. However, only age as a between-person predictor was associated with valence (*p =* .036). We found no significant effects for length of bouts, sex, and day of week. Moreover, we found significant random effects for time, day, social and environmental context, indicating variability between participants. Detailed results are shown in Table 3.

**Table 3:** Multilevel model analyses predicting mood: Fixed and random effects of time [h], time-squared [h^2^], sex, age [yrs], type of study, day, social and environmental context.

Outcome		Fixed effects					Random effects			
	Predictor	beta-coefficient	Standard Error	t-value (df)	P-value	R^**2**^ [%]	Variance Estimate	SD	Wald-Z	P-value
**Model 1: Valence**	Intercept	4.3	0.17	25.19 (418)	< 0.001		0.25	0.05	4.93	< 0.001
	Time	-0.05	0.014	-3.76 (1874)	< 0.001	0.74	0.001	0.0003	3.44	0.001
	Time-squared	0.003	0.001	3.8 (1614)	< 0.001	0.92				
	Length of bouts (30 min)^a^	-0.04	0.09	-0.45 (259)	0.654	< 0.00				
	Day (weekday)^b^	0.05	0.05	0.89 (149)	0.373	0.74	0.09	0.03	3.5	< 0.001
	Age	0.01	0.005	2.14 (258)	0.034	0.72				
	Sex (female)^c^	0.002	0.09	0.02 (249)	0.983	< 0.00				
	Social context (not-alone)^d^	0.17	0.04	4.9 (160)	< 0.001	1.06	0.03	0.01	2.62	< 0.001
	Environmental context (leisure)^e^	0.33	0.05	6.4 (196)	< 0.001	3.69	0.11	0.03	4.07	< 0.001
**Model 2: Energetic arousal**	Intercept	3.64	0.2	18.43 (416)	< 0.001		0.17	0.06	2.69	0.007
	Time	0.04	0.02	2 (2248)	0.046	0.58	0.001	0.0005	3.67	< 0.001
	Time-squared	-0.003	0.001	-3.72 (1948)	< 0.001	0.90				
	Length of bouts (30 min)^a^	-0.1	0.1	-0.99 (235)	0.326	0.08				
	Day (weekday)^b^	-0.16	0.07	-2.21 (197)	0.029	0.32	0.2	0.05	4.51	< 0.001
	Age	0.02	0.01	4.65 (235)	< 0.001	1.51				
	Sex (female)^c^	-0.3	0.1	-3.6 (211)	0.002	0.96				
	Social context (not-alone)^d^	0.24	0.05	4.94 (155)	< 0.001	0.22	0.08	0.03	3.06	0.002
	Environmental context (leisure)^e^	-0.22	0.06	-3.6 (211)	< 0.001	1.37	0.13	0.04	3.63	< 0.001
**Model 3: Calmness**	Intercept	4.29	0.16	27.29 (490)	< 0.001		0.24	0.04	5.59	< 0.001
	Time	-0.06	0.01	-4.4 (3891)	< 0.001	0.07				
	Time-squared	0.003	0.001	4.5 (3863)	< 0.001	0.14				
	Length of bouts (30 min)^a^	0.18	0.08	2.23 (290)	0.026	0.67				
	Day (weekday)^b^	0.08	0.05	1.64 (141)	0.104	0.35	0.06	0.02	2.78	0.005
	Age	0.01	0.004	2.27 (274)	0.024	1.21				
	Sex (female)^c^	0.03	0.08	0.36 (265)	0.719	< 0.00				
	Social context (not-alone)^d^	0.06	0.04	1.69 (166)	0.093	0.18	0.39	0.01	2.79	0.005
	Environmental context (leisure)^e^	0.27	0.05	5.32 (262)	< 0.001	1.72	0.12	0.02	4.87	< 0.001

#### Energetic arousal

Social context significantly predicted energetic arousal (*p* = < .001), i.e., sitting while being with others was associated with higher energetic arousal ratings: being alone compared to being not alone revealed lower ratings of energetic arousal by 0.24 units [scale 1–6]. Moreover, the environmental context significantly predicted energetic arousal (*p* = < .001), i.e., sitting during leisure time was associated with lower energetic arousal ratings. In particular, being at work compared to being at the leisure context increased ratings of energetic arousal by 0.22 units [scale 1–6]. The results also showed that time (*p* = .046) and time-squared significantly influenced energetic arousal (*p* = < .001) in both positive and negative directions. In practice, the reported level of energetic arousal indicated a reversed u-shaped curve, i.e., an increased level until approximately 2 pm followed by a subsequent decrease until the end of the day. Furthermore, the between-person variance (age, sex, and day of week) was significantly associated with energetic arousal (see Table 3). In particular, higher age was associated with higher ratings of energetic arousal (*p* = < .001). Furthermore, females reported lower ratings than males (*p* = .002). Participants reported lower ratings of energetic arousal on weekdays compared to weekends (*p* = .029). We found no significant effect for length of bouts on energetic arousal. Moreover, we found significant random effects for time, day of week, social and environmental context, indicating variability between participants.

#### Calmness

In contrast to our expectations, we found no significant association between social context and calmness ratings (*p* = .093). However, again we found that the environmental context significantly predicted calmness (*p* = < .001), i.e., sitting in the leisure context was associated with higher calmness ratings: sitting at work compared to sitting while pursuing leisure activities decreased calmness ratings by 0.27 units [scale 1–6]. Furthermore, time and time-squared significantly influenced calmness (all *p’s* = < .001) in both negative and positive directions. Calmness decreased during the day until approximately 3 pm, followed by a subsequent increase until the end of the day (see Table 3). Our results also revealed that age and length of bouts were significantly associated with ratings of calmness. In particular, higher age was associated with higher calmness ratings (*p* = .033), and sedentary bouts of ≥30 min differed significantly (*p* = .026) compared to 20 min sedentary bouts by 0.19 units. We found no associations between sex and weekday and ratings of calmness. Moreover, we found significant random effects for day, social and environmental context, indicating variability between participants.

### Exploratory interaction analyses

In each of the previously presented models, the interaction of social and environmental contexts was integrated. Thus, resulting in four conditions with the following number of cases: alone in the leisure context (*n* = 656), alone at work (*n* = 1275), not alone in the leisure context (*n* = 1072); and not alone at work (*n* = 1238). In two of three affect dimension models, the added interaction effect significantly improved model fit compared to the previously reported models. In particular, the model fit of the outcome valence (−2ΔLL(1) = 20.8, p = < .001) and energetic arousal (−2ΔLL(1) = 39.7, *p* = <.001) improved but not of the outcome calmness (−2ΔLL(1) = 0.8, *p* = .379). Moreover, the interaction effect accounted for an additional 0.42, 0.45, and 0.12% of the total variance in valence, energetic arousal, and calmness, respectively. The significant interaction effect between social and environmental context on valence (β = 0.32; t (df) = 4.96 (1741); *p* = <.001), energetic arousal (β = 0.54; t (df) = 6.63 (1713); *p* = < .001) and calmness (β = 0.13; t (df) = 2.21 (3949); *p* = .027) indicates that sitting while being with others in the leisure context is related to higher ratings in all three affect dimensions compared to other conditions (i.e., being alone at work, alone in the leisure context or not-alone at work).

## Discussion

To advance guidelines and to create effective intervention concerning SB, we need to identify those sedentary episodes that are negatively associated with mental health. In this study, we combined four studies, which assessed SB objectively with a thigh-worn accelerometer and which used a sedentary-triggered E-diary to capture context data during prolonged sitting bouts. Participants showed on average five prolonged sitting bouts during one day, which is consistent with recent studies [4, 40]. During these prolonged sitting bouts they felt, on average, well (valence), relaxed (calmness) and slightly energized (energetic arousal). The ICCs indicated that approximately two-thirds of the affect-variances were caused by within-subject variations (e.g. due to situational effects during prolonged sitting). To estimate these situational effects, we compared the dimensions of affective states in two different social contexts (not-alone vs. alone) and in two different environmental contexts (leisure vs. work). Concerning social contexts, participants felt more well and content (valence) as well as more energized (energetic arousal) when sitting while being together with others compared to sitting while being alone. Calmness was not significantly predicted by social context. This finding is in line with a current EMA-study with 108 older adults (*M*_age_ = 72 years), in which the social context (alone vs. not alone) moderates the association between SB and negative affect [41].

Several theoretical assumptions in the field of (social-) psychology as well as empirical evidence highlighted that having contact with others is associated with higher levels of (affective) well-being [42, 43]. For instance, an AA with 245 undergraduates was conducted to examine whether affective states differed depending on whether individuals were alone or with others in daily life [44]. Participants were asked eight times a day for seven days. Participants generally reported more positive and less negative affect during situations in which they were together with others than when they were alone. Another AA study included data of 64,213 social interactions from 150 adults across nine weeks. The study analyzed how different social context factors (e.g. familiarity, type of social partner, gender composition) are associated with affective states in daily life. Results showed that interactions with family members or friends were associated with more positive valence whereas nonfamily social partners were associated with higher ratings of arousal. Therewith, our findings are in line with the well-documented effect that individuals who are together with others report higher levels of positive affective states, which is again associated with improved mental health [45]. Although this finding is not surprising, it extended existing research about the effects of prolonged sitting bouts: Dependent on the social context, prolonged sitting bouts could positively be associated with affective states and therewith with mental health.

The second main effect is related to environmental contexts of leisure and working. Participants felt less well (valence), more energized (energetic arousal) and less calm (calmness) while sitting during work compared to sitting during leisure time. This finding is exactly in line with the results of a former AA-study with 87 undergraduates’ students, who were asked every 45 min during one 14-h daytime period [46]. It seems reasonable that subjects felt better and calmer during leisure episodes, because participants could organize their leisure time more autonomously and could therefore do activities, which are more enjoyable and comfortable. It could also be that prolonged sitting bouts during leisure time represents several situations of relaxation and recreation (e.g. watching TV, reading), which are often not associated with feelings of energy.

An interesting result revealed our exploratory interaction analyses: for the affect dimensions valence and energetic arousal the interaction term of social and environmental context was significant and improved model fit. Participants felt best while sitting during leisure time together with others, compared to all other conditions (sitting during leisure and alone, sitting during working episodes and alone vs. not alone). Thus, not all prolonged sitting bouts are equally associated with affective states. Prolonged sitting during leisure time while being together with others are associated with more positive affect compared to other prolonged sitting episodes.

This is relevant for interventions and guidelines to reduce SB. Although presumably not positive for physical health, the results indicate that not all sedentary bouts are detrimental in terms of affective states. Some SB bouts might be related to behaviors/activities (such as talking with friends) that are positively related to momentary affect and might therefore be relevant for social and psychological health. Thereby, it is not necessarily sitting itself that matters, especially in terms of affective states, but what the person is doing and with whom. In light of this, the context of sitting might serve as a proxy for the meaning and purpose of activities undertaken while sitting. In line with Gardner and colleagues [31] the results highlight that reserachers should to shift the attention from sitting itself to what people do while sitting. However, to inform the development of interventions and guidelines, more research is needed that focuses on the variability of sitting contexts - and especially of behaviors that take place while sitting - and the relation to health-related outcomes. To gather deeper insights of the behavior-affect link, which may depend on the context of the behavior, sophisticated approaches such as mixed sampling strategies (e.g., sedentary and activity triggered e diaries) would allow to tackle potential mediation effects between physical behavior, context and affective states. Although this study used an innovative sedentary-triggered AA approach to capture social and environmental context data exactly while participants were sitting more than 20 min, there are several limitations worth noting. First, we did not assess the quality of the social relationship. It could be that this quality moderates the association between social context and affective states. Second, we cannot exclude residual confounds (e.g., those due to other everyday life factors that influence affective states such as nutritional behaviors, partnership quality, employment status, quantity and quality of sleep, and drug consumption such as alcohol and caffeine). Third, our observational study does not prove causality because third confounders might show similar time-related characteristics. Therefore, additional studies are needed, e.g., to conduct “social or environmental experiments” in everyday life. Fourth, we cannot generalize our findings to other populations, thus we emphasis future research endeavors to replicate our findings in different sample or target groups such as adolescents, elderlys or individuals with other culture backgrounds, for whom social interactions might be more or less important for mental health. Fifth, although our used algorithm detected over 80% of all prolonged sedentary bouts, challenges should be acknowledged, such as technical stability (e.g., accelerometer and the smartphone losing their BLE connection) or user compliance (e.g., not answering the context questions) [32].

## Conclusions

The findings of this study showed substantial within-subject variations of momentary affective states during different prolonged sitting bouts and indicated that participants felt better during situations in which they are together with other people (higher levels of valence and energetic arousal compared to being alone) and during leisure time (with higher levels of valence and calmness and lower levels of energetic arousal compared to work). The results deepened the understanding of the association between momentary affect and prolonged sitting bouts. They reflect that not all prolonged sitting bouts are equally associated with momentary affect. The behavior-affect link depends on the context of the behavior, which might be a proxy for what the person is doing and with whom. These findings are important for SB reducing interventions and could inform the development of behavior change strategies. The knowledge regarding diversity of sitting bouts (related to contexts and behaviors) and their different relation to momentary affect, leads a) to decisions, which sitting bouts should be targeted by the intervention and b) to adapt behavior change strategies to the targeted sitting episodes.

## Supplementary Information

**Additional file 1.**

## Data Availability

The datasets used and/or analysed during the current study are available from the corresponding author on reasonable request.
