# Supplementary material for: The context matters - not all prolonged sitting bouts are equally related to momentary affective states: an ambulatory assessment with sedentary-triggered E-diaries
Source: Int J Behav Nutr Phys Act. 2021 Aug 14;18:106. doi: 10.1186/s12966-021-01170-3 (PMC8364093; doi:10.1186/s12966-021-01170-3)
Supplement: Supplementary file 1 — Additional file 1. [file 12966_2021_1170_MOESM1_ESM.pdf]

Additional file 1

Model 1:

MIXED Valence with tis tiss age by sex bouts_length weekday environmental_context social_context

/CRITERIA=CIN(95) MXITER(100) MXSTEP(10) SCORING(1) SINGULAR(0.000000000001) HCONVERGE(0, ABSOLUTE) LCONVERGE(0, ABSOLUTE) PCONVERGE(0.000001, ABSOLUTE)

/FIXED= tis tiss age bouts_length sex weekday environmental_context social_context | SSTYPE(3)

/METHOD=REML

/RANDOM=INTERCEPT environmental_context scoial_context tis weekday | SUBJECT(Participant) COVTYPE(VC)

/PRINT=G SOLUTION TESTCOV.

Model 2:

MIXED Energeticarousal with tis tiss age by sex bouts_length weekday environmental_context scoial_context

/CRITERIA=CIN(95) MXITER(100) MXSTEP(10) SCORING(1) SINGULAR(0.000000000001) HCONVERGE(0, ABSOLUTE) LCONVERGE(0, ABSOLUTE) PCONVERGE(0.000001, ABSOLUTE)

/FIXED= tis tiss age bouts_length sex weekday environmental_context scoial_context | SSTYPE(3)

/METHOD=REML

/RANDOM=INTERCEPT environmental_context scoial_context tis weekday Study_ID | SUBJECT(Participant) COVTYPE(VC)

/PRINT=G SOLUTION TESTCOV.

Model 3:

MIXED Calmness with tis tiss age by sex bouts_length weekday environmental_context scoial_context

/CRITERIA=CIN(95) MXITER(100) MXSTEP(10) SCORING(1) SINGULAR(0.000000000001) HCONVERGE(0, ABSOLUTE) LCONVERGE(0, ABSOLUTE) PCONVERGE(0.000001, ABSOLUTE)

/FIXED= tis tiss age bouts_length sex weekday environmental_context scoial_context | SSTYPE(3)

/METHOD=REML

/RANDOM=INTERCEPT environmental_context scoial_context weekday | SUBJECT(Participant) COVTYPE(VC)

/PRINT=G SOLUTION TESTCOV.
